# Ebola Virus Infection Associated with Transmission from Survivors

**DOI:** 10.3201/eid2502.181011

**Published:** 2019-02

**Authors:** Saskia Den Boon, Barbara J. Marston, Tolbert G. Nyenswah, Amara Jambai, Moumie Barry, Sakoba Keita, Kara Durski, Schabbethai S. Senesie, Devin Perkins, Anita Shah, Hugh H. Green, Esther L. Hamblion, Margaret Lamunu, Alex Gasasira, Nuha O. Mahmoud, Mamadou H. Djingarey, Oliver Morgan, Ian Crozier, Christopher Dye

**Affiliations:** World Health Organization, Geneva, Switzerland (S. Den Boon, K. Durski, D. Perkins, A. Shah, O. Morgan, I. Crozier, C. Dye);; Centers for Disease Control and Prevention, Atlanta, Georgia, USA (B.J. Marston, S.S. Senesie, H.H. Green, O. Morgan);; National Public Health Institute, Monrovia, Liberia (T.G. Nyenswah);; Ministry of Health and Sanitation, Freetown, Sierra Leone (A. Jambai);; Ministry of Health, Conakry, Guinea (M. Barry, S. Keita);; World Health Organization, Monrovia, Liberia (E.L. Hamblion, A. Gasasira, N.O. Mahmoud);; World Health Organization, Freetown, Sierra Leone (M. Lamunu);; World Health Organization, Conakry (M.H. Djingarey); University of Oxford, Oxford, UK (C. Dye)

**Keywords:** Ebola virus disease, viruses, West Africa, sexual transmission, viral persistence, survivors, surveillance, response, Guinea, Liberia, Sierra Leone

## Abstract

Ebola virus (EBOV) can persist in immunologically protected body sites in survivors of Ebola virus disease, creating the potential to initiate new chains of transmission. From the outbreak in West Africa during 2014–2016, we identified 13 possible events of viral persistence–derived transmission of EBOV (VPDTe) and applied predefined criteria to classify transmission events based on the strength of evidence for VPDTe and source and route of transmission. For 8 events, a recipient case was identified; possible source cases were identified for 5 of these 8. For 5 events, a recipient case or chain of transmission could not be confidently determined. Five events met our criteria for sexual transmission (male-to-female). One VPDTe event led to at least 4 generations of cases; transmission was limited after the other events. VPDTe has increased the importance of Ebola survivor services and sustained surveillance and response capacity in regions with previously widespread transmission.

The 2014–2016 outbreak of Ebola virus disease (EVD) in West Africa was the largest since the discovery of the Ebola virus (EBOV) in 1976 (*1*). More than 28,000 cases and 11,000 deaths were reported from the 3 most affected countries (Guinea, Liberia, and Sierra Leone) (*2*). An unprecedented number of persons survived; >4,500 of the estimated total of >10,000 survivors have been registered in the 3 countries (*3*).

EVD survivors often have substantial long-term medical sequelae (*4*), and viable EBOV can persist in immunologically protected body sites, such as the male gonads and the chambers of the eye (*5**,**6*). Viral persistence in body fluids, such as semen, creates the potential for transmission and initiation of new chains of transmission weeks or months after continuous community transmission has ended (*7*). On January 14, 2016, the World Health Organization (WHO) declared that human-to-human transmission had been stopped in West Africa. However, WHO and its partners indicated that the 3 affected countries remained at high risk for additional EVD outbreaks because of virus persistence in survivors and emphasized the need for strong surveillance and response systems (*8*). A new EVD case was confirmed in Sierra Leone the following day (*9*), and another outbreak comprising cases in both Guinea and Liberia was recognized in March 2016 (*2**,**10*).

Infectious virus has been isolated from semen of Ebola survivors 82 days after symptom onset (*11*), and EBOV RNA has been detected by reverse transcription PCR (RT-PCR) in semen 531 days after symptom onset (*10*). Other body fluids, such as vaginal fluids, breast milk, urine, feces, sweat, and saliva, also have been shown to be RT-PCR–positive for short periods after recovery of the patient (*11*). Some evidence indicates that transmission could occur through breast milk (*12*), but confirmed transmission from viral persistence in the other body fluids has not been described (*13*). Before the West Africa EVD outbreak, 1 case of Marburg disease (*14*) and 1 possible case of EVD (*15*) attributed to sexual transmission had been reported. From the EVD outbreak in West Africa, several incidents of possible sexual transmission of EBOV from EVD survivors have been described in detail (*10*,*16**–**19*), as have viral persistence–derived transmission of EBOV (VPDTe) events for which the mode of transmission was unknown (*12**,**20**–**22*).

We describe a series of EBOV transmission events with evidence of transmission related to viral persistence in EVD survivors. Our findings are relevant for response planning, especially related to surveillance and response capacity, and for the development of policies and guidelines regarding survivor counseling, care, and management.

## Methods

We defined VPDTe as the person-to-person transmission of EBOV from an EVD survivor (source) to another person (recipient) that occurred >21 days after the source case recovered from acute infection. The WHO definition of recovery is >3 days without any fever or symptom and negative blood RT-PCR for EBOV; however, this documentation was not available for all patients (*23*). The 21-day period reflects the upper limit of the incubation period for EVD; incorporating this period into the definition reduces the likelihood that the recipient was not infected during the period of acute infection of the source. The recipient is the person who becomes infected as a result of VPDTe. A recipient might or might not be the first person (index person) detected in a newly identified cluster of EVD cases (and in some situations, clearly identifying the recipient might not be possible). We defined confirmed, probable, and suspected cases of EVD and EVD survivors in accordance with WHO definitions (*24*).

We focused our identification of VPDTe events on the period after control of the most intense periods of transmission in the affected countries and retrieved information about possible VPDTe events identified after February 19, 2015 (after the initial interruption of ongoing transmission in Liberia). During this time, low case counts facilitated recognition and subsequent in-depth case investigation of new EVD cases that were not clearly related to continuous disease transmission in the community and might have resulted from exposure to a survivor with viral persistence. In September 2015, we began to maintain a formal list of suspected VPDTe events identified by staff involved in epidemiologic investigations in the 3 countries. The data were entered retrospectively or prospectively into the formal list.

For all events, the field teams collected information at the time the case or cluster of cases was detected. We extracted data from available databases, WHO Situation Reports, region- and country-specific situation reports, email correspondences to the WHO Ebola Response Information Management team, case investigation reports, transmission chains (including the number of additional cases and generations in the cluster), and genetic sequencing results. We captured demographic data; dates of symptom onset, diagnosis, discharge, and death; laboratory results; epidemiologic investigation details; sequencing data; data on how the cases were initially recognized; information on encounters with healthcare providers; details of outbreak control efforts; and information about the secondary cases.

We developed criteria to determine which transmission events qualified as resulting from viral persistence (Table 1). We also developed criteria for source of transmission (Table 2), mode of transmission, and the strength of the evidence for transmission (strong, moderate, or weak) (Table 3). If the investigation identified >1 possible mode of transmission, these criteria for strength of evidence and evaluation of the route of transmission were not tabulated. Two investigators (S.D., B.J.M.) independently reviewed and classified each transmission event, and a third (S.S.S.) resolved differences.

**Table 1:** Criteria for the qualification of Ebola virus persistence–derived transmission event*

Criteria no.	Explanation
1	Sequencing provides links to a case or cases that occurred >21 days before the index person and not to a more recent case
	OR
2	Absence of known exposure of the presumed recipient (or index person) to a person with Ebola virus disease in the 21 days before infection
	AND
	No evidence of ongoing transmission in the community where case or cluster was recognized
	AND
	Epidemiologic link to survivor established

*Criteria apply to recipient or >1 person in the cluster (if the index person is not the recipient).

**Table 2:** Strength of evidence and criteria for source person for Ebola virus disease*

Strength of evidence	Criteria
Strong, A + B + C	A. Epidemiologic link between index person or first case and single proposed/probable source established
	AND
	B. EBOV RNA detected in a specimen taken from the proposed/probable source after recovery
	AND
	C. Sequencing indicates linkage to virus recovered from recipient or index person
Moderate, A + B or A + C	A. Epidemiologic link between recipient or index person and proposed/probable source established
	AND
	B. EBOV RNA detected in a specimen taken from the proposed/probable source after recovery
	OR
	C. Sequencing indicates linkage to virus recovered from recipient or index person
Weak	Epidemiologic link between recipient or index person and proposed/probable source established
Not identified	No epidemiologic link between recipient or index person and proposed/probable source could be established

*Results of application of these criteria were included in the Appendix Table only when a single possible transmission scenario emerged after case investigation and review. EBOV, Ebola virus.

**Table 3:** Strength of evidence and criteria for sexual transmission of Ebola virus*

Strength of evidence	Criteria
Strong, A + B + C	A. Epidemiologic investigation identified sexual contact between recipient or index person and single proposed/probable source
	AND
	B. EBOV detected in single proposed/probable source’s semen or vaginal secretions (by a vaginal swab) by RT-PCR
	AND
	C. Sequencing indicates high likelihood of transmission to recipient
Moderate, A + B or A + C	A. Epidemiologic investigation identified sexual contact between recipient or index person and single proposed/probable source
	AND
	B. EBOV detected in single proposed/probable source’s semen or vaginal secretions (by a vaginal swab) by RT-PCR
	OR
	C. Sequencing indicates high likelihood of transmission to recipient
Weak	Epidemiologic investigation identified sexual contact between recipient or index person and single proposed/probable source†
None	No report of sexual contact

*EBOV, Ebola virus; RT-PCR, reverse transcription PCR. †If not countered by sequencing data suggesting that the sexual contact is unlikely to be the source.

The WHO Ethics Review Committee (ERC.0002736) approved this activity, and the Centers for Disease Control and Prevention classified it as a nonresearch program activity. The analysis of these surveillance data from the EVD epidemic was approved by representatives of the Ministry of Health in each of the 3 countries and was not considered to require additional ethics review. In accordance with the approved protocols, we have omitted information that would enable identification of specific persons.

## Results

We identified 13 transmission events that met criteria for VPDTe during the evaluation period (Appendix). We identified a recipient for 8 events; among these, 7 were female. Ages of all recipients ranged from 16 to 55 years. For the remaining 5 events, the recipient was not clearly identified, and the first person identified in the cluster might or might not have been the recipient. For these VPDTe events, we report details for the first person identified. Eight of the 13 events have previously been described separately in the context of outbreak investigations (*10**,**12**,**16**–**22*).

### Description of VPDTe Events Not Previously Described Elsewhere

#### Event 1

Postmortem testing determined the recipient in this event had EVD. No cases had occurred in the immediate area during the 7 weeks before the report of the index person. Although initial epidemiologic investigation suggested possible exposures through funeral attendance, viral sequencing suggested a link to an EVD cluster that had occurred in a nearby geographic area ≈7 weeks earlier. That cluster included 1 male survivor; he reported sexual contact with the recipient. A semen sample from the survivor tested positive for EBOV RNA by RT-PCR. Sexual contact was considered the likely route of transmission.

#### Event 5

The recipient in this event died in the community the same day as symptom onset; EVD was confirmed by testing of a postmortem oral swab sample. Based on genetic sequencing, the virus from the recipient did not group with ongoing chains of transmission but did group with sequences from viruses identified earlier in the epidemic. Epidemiologic investigations showed the recipient had had sexual contact with a male EVD survivor 2 months after his discharge from an Ebola treatment unit and ≈1–3 weeks before she became symptomatic. Semen from the survivor was not tested, but EBOV RNA from a stored blood sample collected during the survivor’s initial illness showed genetic sequence that was similar to that of the recipient.

#### Event 6

In this event, a young woman with symptoms of EVD sought care at a healthcare facility, and EVD was confirmed by RT-PCR. Epidemiologic investigation found no history of travel, funeral attendance, or bush meat consumption. There was no known ongoing transmission in the village or district. Sequences of viral RNA from the recipient clustered with isolates from cases that occurred in the same area 5–11 months earlier but differed substantially from available individual sequences from persons with contemporaneous confirmed EVD. The route of transmission is unknown.

#### Event 8

In this event, a man had EVD confirmed based on a swab specimen collected after his death. Sequencing linked his virus to viruses from cases 5 months earlier in a different district. No specific epidemiologic links to recent or more remote cases were identified. The source and route of transmission are unknown.

#### Event 9

This event involved EVD reported in a woman 2 days after her death and confirmed by testing of a postmortem oral swab sample. Her viral sequence did not match contemporary circulating strains of EBOV but did link to strains from ≈5–6 months earlier. Her husband spent time in both the community where she lived and in the capital city; he had experienced an illness compatible with EVD 5–6 months earlier but had not been tested. Two other persons had died recently in the family (a co-wife and the child of the co-wife), and a stillbirth occurred (to the niece of the index person); no testing for EVD had been conducted in these situations. Transmission had not been recognized in the community before the recognition of this cluster of illness, and there was no known exposure to an ill person or funeral. The source and route of transmission are unknown. One possible explanation is that the husband was an EVD survivor and had transmitted EBOV through sexual or other contact to >1 members of the family; direct transmission also might have occurred from close contact.

### Mode of Transmission and Strength of Evidence

For 4 events, we could not identify a single clear hypothesis for how transmission occurred. All 4 of these have been described in separate publications (*12*,*16*,*21*,*22*). Five events met our defined criteria for sexual transmission (Appendix Table). The evidence was strong for 4 events and moderate for 1 event. Among these 5 VPDTe events of suspected sexual transmission, we identified the survivor believed to be the source; all were male. The other 8 VPDTe transmission events for which the mode of transmission is unknown were considered to be due to survivor transmission because they were isolated cases and sequencing results linked them to previous transmission chains. The routes of transmission are unknown.

### Detection and Response

In 10 VPDTe events, EBOV infection was diagnosed in the index person on the basis of a postmortem sample, or it was preceded by deaths that met the definition for probable EVD; ultimately, all but 1 of the index persons or recipients of VPDTe died. Eight recipients had reported encounters with health providers before being tested for and receiving a diagnosis of EVD. For 6 VPDTe events, cases occurred in addition to the index person (range 1–12 cases; Appendix Table). Most clusters were limited to 0 or 1 additional generation of cases, but in 1 cluster, several additional generations occurred.

After detection of the index person, response activities such as isolation and management of the index patient and contact tracing with isolation of high-risk and symptomatic contacts were initiated for all events. Seven cases occurred after initial control of ongoing transmission in a specific country and required reactivation of some response resources. Searches for missing contacts were intense and continued until at least the end of the initial 21-day follow-up period. For some clusters, there were difficulties engaging with local communities, complicating contact tracing. For at least 8 events, Ebola vaccine was provided to contacts and to contacts of contacts under research protocols and emergency use licensure.

## Discussion

On March 29, 2016, WHO declared the end of the Public Health Emergency of International Concern regarding the EVD outbreak in West Africa (*25*). In the declaration, WHO emphasized that new EVD clusters would continue to occur because of reintroductions of EBOV from survivors. Our case series summarized 13 such events.

Most of the events we describe were initially recognized because they occurred in isolation, in areas that EBOV had not previously affected, or well after community transmission had ended. Plausible explanations for such cases include recrudescence of prior EVD, reintroduction from a zoonotic reservoir, transmission resulting from unrecognized contact with a person with active EVD, or transmission related to viral persistence. Recrudescence might have played a role in event 11, but EVD was not confirmed in the mother of the index person at the time of her initial EVD-like illness, and there was no documentation that she was shedding EBOV in the period preceding illness in the index person (*22*). We do not believe any of the other events described here occurred because of EBOV recrudescence. Based on sequencing data, the cases we describe were genetically related to other cases that occurred as part of human-to-human transmission in the West Africa epidemic; no evidence exists to indicate these cases resulted from reintroduction from a zoonotic reservoir. Given the absence of additional recognized transmission events, we believe it highly unlikely that the cases we summarized were part of undetected transmission chains.

Routes of VPDTe are not fully understood. We found evidence of sexual transmission in at least 5 of the events; sexual transmission might have played a role in additional events, but we cannot exclude other modes of VPDTe. EBOV was found in the breast milk of a woman in 1 cluster; transmission through breast milk might have occurred in this event.

We are unable to quantify the risk for VPDTe over time. There have been a limited number of cases of recognized sexual transmission, even though >10,000 persons are estimated to have survived (*3*). The number of male survivors who have EBOV persisting in semen declines over time, reducing the likelihood of events caused by sexual transmission (*26*). Transmission because of viral persistence in body fluids other than semen might occur but probably less frequently (*3**,**12**,**13*).

WHO’s interim advice on the sexual transmission of EBOV is that male EVD survivors should continue to practice safer sex (i.e., correct and consistent condom use or abstinence) for at least 12 months after symptom onset or until their semen has twice tested negative (*27*). This case series highlights 2 events of VPDTe that occurred >1 year after recovery by the source case. This information, along with information about the duration of persistence of EBOV RNA in the semen of survivors and data showing that EBOV RNA can be detected intermittently (*7*,*28*), needs to be considered in the development of recommendations for EVD survivors. Furthermore, recommendations on semen testing and safer sex practices, including timely updates if recommendations are revised in light of new evidence, must be clearly communicated to EVD survivors and to the public.

Ideally, counseling and testing should be offered as part of a comprehensive package of care for survivors that recognizes the challenges faced by survivors, including health problems, mental health problems, rejection, and stigma (*29**–**33*). Furthermore, partners and family members of EVD survivors need to be counseled, and community education about VPDTe needs to be provided.

An important element of surveillance for EVD is that early recognition of isolated cases is difficult. Despite the context of a recently controlled enormous EVD epidemic, most of the VPDTe cases were recognized very late in illness or after death. This finding speaks to the nonspecificity of EVD symptoms; the high prevalence of other diseases with similar symptoms; and the limited resources for identifying the specific cause of disease in low-resource settings, such as West Africa. Wider availability of diagnostic testing, for example with rapid tests, may support more timely diagnosis than would otherwise have been possible.

One new case of EVD could potentially result in large numbers of secondary cases (*34*). Therefore, it is critical to sustain the capacity to respond rapidly, particularly after an EVD outbreak, including the capacity to conduct outbreak investigation and trace contacts; efficient collection, transport, and laboratory diagnosis of specimens; isolation and clinical management of cases; and, as appropriate, vaccination of contacts and contacts of contacts.

The responses to the events we describe were generally robust, and transmission was in most instances limited to 1 generation. This observation reflects the control capacity generated in the context of the broad outbreak response, a result of enormous effort on behalf of the governments and ministries of health of the affected countries and immense mobilization of resources from the international community and international partners.

Identifying routes of EBOV transmission is not straightforward. Rigorous epidemiologic investigation and evaluation of viral RNA sequences can provide helpful information. Sequencing helped to confirm the link to an epidemiological source for several cases and for some cases suggested a link that was later confirmed epidemiologically. Given this contribution, we believe that sequencing can play a critical role in efforts to control infectious diseases. Still, for several situations that appeared to involve viral persistence, the route of transmission was unclear, and even when we identified a single likely route of transmission, we could not exclude the possibility that we missed alternate possible routes of transmission.

Our findings are subject to several additional limitations. Specifically, our list of described events is unlikely to include all events of VPDTe. Particularly at the height of the outbreak, distinguishing between direct transmission from active EVD cases and VPDTe was not possible, and we therefore concentrated on describing cases that occurred after control of the most intense periods of transmission in the affected countries. Another study identified 2 additional EVD clusters possibly resulting from VPDTe by using the signature of a reduced evolutionary rate (*21*). Further analysis of sequence data from cases in West Africa might identify additional events of possible VPDTe. In the future, expanded use of sequencing and semen testing could help identify additional events.

Taking these limitations into consideration, the number of events identified here most likely underestimates VPDTe in West Africa. Furthermore, the information and evidence for each event was collected on an ad hoc basis, depending on the characteristics of the particular event. Where available, we collected information about viral sequence using various methods, and we relied on the reports and interpretations of sequence data rather than an analysis of the primary data. In some instances, source case documents included conflicting information and the deaths of all but 1 recipient or index person limited the epidemiologic investigations. As a result, information about some of the events might be incomplete; despite intense investigation, in some cases, critical questions remain about how transmission occurred.

Our report has several key implications. EVD cases and clusters might occur after initial control of an EVD outbreak. Although previously available science supported the possibility of viral persistence, the West Africa outbreak clearly illustrated the potential consequences of EBOV persistence in terms of program needs, stigmatization of survivors, and risk that transmission from a survivor with persistent virus will trigger a new cluster. With this new reality, it is critical that the responses to EVD clusters include sustained vigilance, maintenance of response capacity, and continued efforts to reduce the risks that survivors in whom EBOV persists will transmit infection.

Appendix Ebola virus persistence–derived transmission events.
